# Dose–response effects of physical exercise standardized volume on peripheral biomarkers, clinical response, and brain connectivity in Parkinson’s disease: a prospective, observational, cohort study

**DOI:** 10.3389/fneur.2024.1412311

**Published:** 2024-07-03

**Authors:** Rossella Rotondo, Elvira Padua, Giuseppe Annino, Michele Guescini, Sabrina Donati-Zeppa, Michela Goffredo, Vilberto Stocchi, Fabrizio Stocchi, Maria Francesca De Pandis

**Affiliations:** ^1^San Raffaele Cassino, Cassino, Italy; ^2^Department of Human Science and Promotion of Quality of Life, San Raffaele Rome Open University, Rome, Italy; ^3^Center of Space Bio-Medicine, Department of Medicine Systems, Tor Vergata University, Rome, Italy; ^4^Department of Biomolecular Sciences, University of Urbino Carlo Bo, Urbino, Italy; ^5^Department of Neurological and Rehabilitation Sciences, IRCCS San Raffaele Roma, Rome, Italy

**Keywords:** exercise, Parkinson’s disease, rehabilitation, metabolic equivalent of task, neurotrophic actors, peripheral biomarkers, neuroplasticity, brain connectivity

## Abstract

**Background:**

Exercise has been proposed as the “Universal Prescription for Parkinson’s Disease”; however, the specificity of exercise dose in terms of frequency, intensity, duration, and type to be prescribed remains to be elucidated. The 2018 US updated guidelines and WHO Guidelines on Physical Activity and Sedentary Behavior recommend older adults (> 65+ years) to achieve weekly minimal activity levels, indicating the intensity of aerobic exercise as the metabolic equivalent of task and duration as minutes/week (150–300 min/week at a moderate intensity of 3–5.9 MET- or 75–150 min/week of a vigorous intensity of ≥6 MET). Translating these recommendations to PD patients, the study aimed to assess the dose–response effects of standardized volume of structured exercise, measured as METs-minutes/week (weekly energy expenditure) of two different rehabilitation settings to quantify the change in neurotrophic factors. The exercise-induced benefits between the two rehabilitation settings will be evaluated based on motor and non-motor symptoms, kinematic parameters of gait, cognitive function, quality of life, and cortical activity and brain connectivity.

**Methods:**

METEX-PD is a pilot, prospective, observational, cohort study. The study will enroll consecutively thirty (*N* = 30) participants with mild-to-moderate Parkinson’s disease diagnosis to be assigned to a non-intensive or intensive rehabilitation group. The non-intensive rehabilitation group will achieve a range of 180–270 METs-min/week (90 min/week of low-intensity aerobic exercise, 2–3 METs), while the intensive rehabilitation group will exercise at 1350–1980 METs-min/week (225 min/week of high-intensity aerobic exercise, 6–8.8 METs). The METEX-PD trial will last 12 weeks, including 4 weeks of aerobic training program and two follow-ups. Assessments will be performed at baseline (T0), at the end of the exercise program (T1—end of the program), and 4- and 8 weeks after the end of the training program (FU-1 and FU-2). The primary outcome is the change from baseline in peripheral blood BDNF levels. Secondary outcomes are differences in peripheral biomarkers, functional-motor assessments, clinical-functional evaluations, and brain imaging.

**Conclusion:**

METEX-PD trial will enable us to estimate the change in BDNF levels and other peripheral biomarkers under precise exercise-induced energy expenditure. The primary results of the METEX-PD study will allow the development of a larger multicenter randomized controlled trial to investigate the molecular pathways inducing the change in selected neurotrophic factors, such as BDNF, IGF-1, or irisin, and the downstream mechanisms of neuroplasticity in PD patients.

## 1 Background

Over the past two decades, aerobic exercise has emerged as a mainstream recommendation to improve motor function and quality of life in people with Parkinson’s disease (1). Although exercise has been reported as “The Universal Prescription for Parkinson’s Disease,” the specificity of exercise prescription in terms of frequency, intensity, duration, and type to be followed to maximize its beneficial effects remains unclear. Furthermore, the inappropriate use of physical activity and exercise as interchangeable terms contributed to providing a non-specific exercise recommendation to PD patients (2). Following the American College of Sports Medicine (ACSM) Guidance for Prescribing Exercise (3), physical activity is defined *as any bodily movement that produces energy expenditure. Exercise is a subset of physical activity that is planned, structured, and repetitive and has as a final or an intermediate objective for the improvement or maintenance of physical fitness* (4).

To compare doses and frequencies of exercise regimens to PD medications, the efficacy and safety of aerobic exercise are being tested in the clinical pipeline. Indeed, similar to pharmacological trials evaluating the safety of escalating drug dosages in Phase 2, and determining the efficacy of different dosages of medications in Phase 3, two main trials assessing safety (SPARX2) and efficacy (SPARX3) of moderate-intensity vs. high-intensity aerobic exercise in *de novo* PD patients have been conducted (5).

Since the results of the SPARX2 trial proved the non-futility threshold by high-intensity but not by moderate-intensity exercise compared with the control group, the ongoing SPARX3 trial is evaluating the efficacy of high-intensity endurance exercise to attenuate the progression of motor signs in *de novo* PD patients, measuring the change in Movement Disorders Society—Unified Parkinson’s Disease Rating Scale (MDS-UPDRS) motor score (part III) (6).

The MDS-UPDRS motor score is generally used to assess the disease-modifying effects of pharmacological treatments in PD. Similar to pharmacological trials, the MDS-UPDRS motor score was adopted as the primary outcome in the SPARX3 trial. However, change in this primary outcome cannot explain the molecular mechanisms driving neuroplasticity changes in PD patients performing exercise.

Several animal studies (7–15) and human trials (16, 17) suggested that aerobic exercise facilitates structural and functional changes in the CNS, potentially involving neuroprotective and neuroplasticity phenomena (18, 19).

Pioneering studies in this field have revealed the neuroprotective effects of exercise in PD rodent models. In particular, Zigmond et al. (10) showed that exercise performed before toxin treatment reduced the dopaminergic neurotoxin-induced behavioral impairments and the loss of dopaminergic neurons as assessed by PET imaging, biochemical, or histochemical assays of tissue samples. The authors suggested the involvement of glial-derived neurotrophic factor (GDNF)-mediated signaling cascade to trigger endogenous neuroprotective effects on dopaminergic neurons. The increase in brain-derived neurotrophic factor (BDNF) and GDNF levels was also observed in Tajiri et al. (14), who compared the effects of running wheel exercise with respect to sedentary behavior in PD rat models of the striatal unilateral lesion with 6-hydroxydopamine (6-OHDA). Therefore, several authors suggested that the neuroprotective effects of exercise in PD animal models were mediated by neurotrophic, anti-inflammatory, and angiogenic factors (7, 9, 14, 20–23). Indirect evidence supports the neuroprotective role of exercise also in people with PD, potentially involving changes in neurotrophic and anti-inflammatory factors (18, 19, 24).

Among neurotrophic factors (NFs), the attention was primarily focused on BDNF, which plays a pivotal role in neurogenesis, synaptic transmission, and plasticity in the hippocampus, acting as a key regulator for the long-term potentiation (LTP), learning, and memory (25). Several animal studies demonstrated that the activation of the BDNF signaling cascade is crucial to observing the exercise-induced effects on hippocampal plasticity (26–28).

However, recent systematic reviews and meta-analyses, which included randomized and non-randomized controlled trials (RCTs and no-RCTs), revealed that a dose–response relationship between exercise and BDNF release needs to be further clarified (18, 19).

Indeed, a few low-risk-of-bias randomized controlled trials (RCTs) investigated aerobic exercise-induced changes in NFs and plasticity-related mechanisms. The first evidence of Frazzitta et al. (29), which linked the improvements in motor signs with an increase in serum BDNF levels in people living with PD performing a moderate-intensity aerobic exercise (≤60% HRR, treadmill), was reinforced by the results of Szymura et al. (30). The latter not only showed a significant increment in serum BDNF concentration but also in β-neurotrophic factor ( β -NGF) in people with PD performing a moderate-intensity aerobic exercise (60–70% HR_max_, balance training). However, the study did not reveal any significant impact on IGF-1 concentrations in the same conditions. To understand the contribution of aerobic and anaerobic exercises to BDNF release, O’Challagan et al. (31) compared the effects of high-intensity interval training (HIIT, ≥85% HR_max_, speedflex machine) with a moderate-intensity resistance and aerobic exercise (60–80% HR_max_, treadmill). This study revealed a significant increase in serum BDNF levels from the first to the last session for the HIIT intervention but not for the moderate-intensity continuous training (MICT). The contribution of exercise complexity to BDNF release was further explored by Freidle et al. (32), who incorporated goal-based training in a homemade aerobic program, but without positive achievements.

Considering the high heterogeneity within training programs performed by individuals with PD in clinical trials, we conducted a systematic review and meta-analysis coding the different training programs of studies included in accordance with Zhou et al. (33) and generalized our findings as physical activity-induced changes in NFs rather than as changes induced by exercise (19).

Our study revealed limited evidence to support or refute the increase in serum/plasma concentration of neurotrophic factors (NFs), BDNF, and insulin-like growth factor (IGF-1) in people with PD performing physical activity (19). Therefore, the need to develop a rigorous clinical trial, with a standardized volume of physical exercise to establish a dose–response relationship between structured exercise and NF levels, may contribute to changes in brain connectivity in people living with PD.

In this regard, a recent systematic review by Li et al. (33) revealed that exercise-induced neuroplastic effects were mediated by increased activation and network connectivity toward normal function or improving the efficiency of compensatory brain networks (17, 34–36), rather than by inducing alteration in gray matter volume (33, 36). However, a direct correlation between BDNF release under a predeterminate dose of exercise and changes in brain connectivity in people with PD has not yet been established.

We designed a pilot prospective observational clinical trial to evaluate the dose–response effects of two different rehabilitation settings characterized by different workloads (measured as energy expenditure) on neurotrophic factors and clinical symptoms.

Energy expenditure *is defined as the total amount of energy (gross) expended during exercise, including resting energy expenditure (resting energy expenditure + exercise energy expenditure)* (3). Since energy expenditure may be articulated in the metabolic equivalent of task (MET), the volume of exercise can be expressed as METs-minutes/week, thus considering the frequency, intensity, time, and type (FITT) of each session of structured exercise. By convention, 1 MET is equal to an oxygen uptake of 3.5 mL∙kg^−1^∙min^−1^ (3).

**Figure 1:**
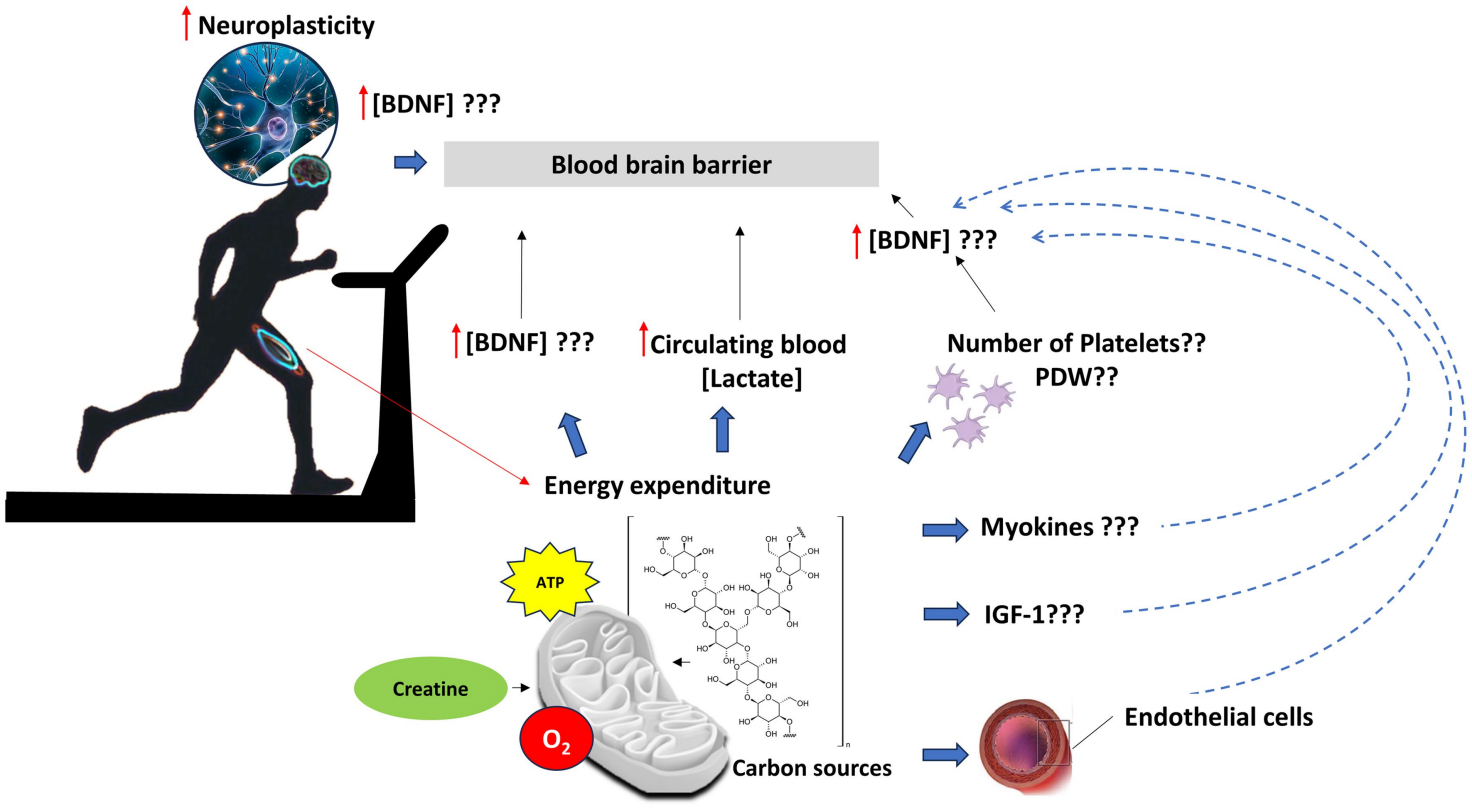
Potential biochemical pathways orchestrating neuroplasticity process in PD patients performing standardized volume of physical exercise. Muscle work needs energy, thus much oxygen consumption to generate ATP, through aerobic and anaerobic processes, by which energy-rich macromolecules (such as glycogen) are transformed into less energy compounds (lactate, H_2_O, CO_2_, and creatine). Skeletal muscle contraction and related energy expenditure can increase concentrations of BDNF levels in the central nervous system, through direct and indirect mechanisms. The release of myokines, such as irisin, may induce BDNF through a PGC-1α/FNDC5 (38) pathway in the hippocampus; energy consumption to sustain muscle contraction induces an increase in circulating blood lactate, which induces the *bdnf* gene expression and TRKB signaling in the hippocampus via NAD-dependent deacetylase sirtuin-1 (SIRT1). In turn, SIRT1 increases the levels of the PGC-1α/FNDC5 pathway and thus the *bdnf* gene expression (39). Peripheral increase could be also the result of BDNF release from platelets, the major storage of pro-BDNF, which is supposed to be produced in megakaryocytes (40–42). The storage of BDNF in the platelets may be correlated with their number and platelet distribution width (PDW). Moreover, exercise can induce a rapid release of peripheral circulating levels of IGF-1, which, in turn, can regulate BDNF levels (28). Finally, vascular endothelial cells may contribute to the production, storage, and release of BDNF centrally as well as peripherally, in response to eNOS activity (43).

The intrinsic definition of physical exercise as energy expenditure explains the rationale of the study, which aims to investigate the biomolecular drivers of exercise-induced neuroplasticity phenomena in people with PD. Muscle work needs energy and requires much oxygen consumption to generate ATP. During aerobic and anaerobic processes, the energy-rich macromolecules (carbohydrates, fat, and phosphocreatine) are transformed into less energy compounds (lactate, H_2_O, CO_2,_ and creatine). The authors hypothesize that a molecular choreography during exercise could orchestrate the activation of different biochemical pathways (37) in a dose–response manner, potentially capable of inducing changes in NFs and, therefore, in the brain connectivity of people living with PD (Figure 1).

### 1.1 Objectives

This pilot observational study will evaluate the dose–response relationship between the volume of exercise, measured as METs-minutes/week, of two different rehabilitation settings to quantify the change in neurotrophic factors driving neuroplasticity in PD patients. The study will also compare the changes induced by non-intensive and intensive rehabilitation settings on motor and non-motor symptoms, kinematic parameters of gait, cognitive function, quality of life, and the changes in cortical activity assessed with electroencephalogram (EEG) and in brain connectivity by functional magnetic resonance imaging (fMRI).

## 2 Methods and analysis

### 2.1 Study design and setting

METEX-PD is a monocentric pilot, prospective, observational, cohort study.

This study will be conducted by the research unit of the Clinical Trial Center for Parkinson’s Disease at San Raffaele Cassino Hospital (CTC for PD—SR Cassino), affiliated with the academic center of IRCCS San Raffaele Rome and in collaboration with the San Raffaele University of Rome, the University of Rome Tor Vergata, and the University of Urbino Carlo Bo.

**Figure 2:**
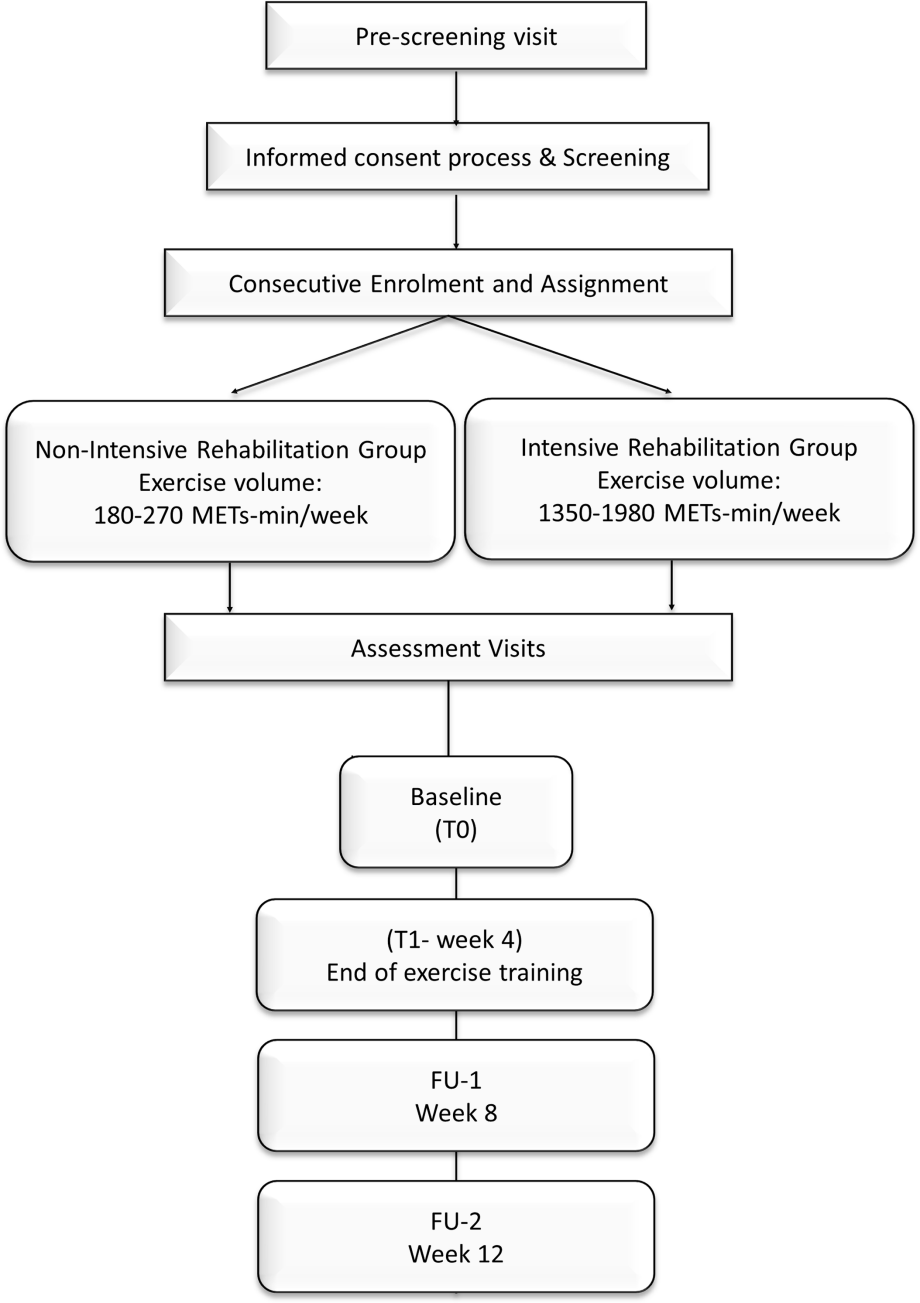
Study design of METEX-PD trial. The non-intensive rehabilitation group assigned will exercise for a range volume of 180–270 METs-min/week, while patients assigned to the intensive rehabilitation group will exercise for 1,350–1980 METs-min/week (44). The duration of the training is 4 weeks. Assessments will be performed at baseline (T0) and the end of exercise training (T1). Follow-up assessments will be scheduled at weeks 8 (FU-1) and 12 (FU-2).

The study will last 12 weeks, including 4 weeks of aerobic training program and two follow-ups. Assessments will occur at baseline (T0), at the end of the exercise program (T1—end of the program), and 4 and 8 weeks after the end of the training program, FU-1 and FU-2, respectively (Figure 2) (31).

### 2.2 Participants

In total, thirty (*N* = 30) participants, who will meet the eligibility criteria, will be consecutively enrolled and assigned to the non-intensive rehabilitation or intensive rehabilitation group. Similar to pharmacological trials, the inclusion/exclusion criteria have been established in accordance with the SPARX2 trial results, which demonstrated the feasibility and safety of high-intensity aerobic exercise in people with PD (5). All patients will be evaluated in the ON phase.

#### 2.2.1 Inclusion criteria

- Diagnosis of Parkinson’s disease according to the United Kingdom (UK) Parkinson’s Disease Society Brain Bank (45)
- Aged between 30 and 80 years.
- Disease stage II-III in the “ON” phase according to modified Hoehn and Yahr (H&Y) (46)
- Having no severe cognitive impairment:

o Mini-Mental State Examination—MMSE ≥24.
o Montreal Cognitive Assessment—MoCA ≥17/30.

- Under stable dopaminergic pharmacological treatment.
- A motor condition that permits to execute 6-min walking test (6MWT).
- Willing to participate in the study, understand the procedures, and sign the informed consent.

#### 2.2.2 Exclusion criteria

- Diagnosis of neurological disorders not related to Parkinson’s disease.
- Musculoskeletal diseases that could impair gait and execution of the exercise program.
- The presence of known cardiovascular disease that can compromise the performance required by the protocol.
- The presence of diabetes or other metabolic and endocrine diseases.
- Uncontrolled hypertension (resting blood pressure > 150/90 mmHg).
- Individuals with orthostatic hypotension and systolic pressure in feet below 100 will be excluded. Orthostatic hypotension (OH) is a reduction in systolic blood pressure of at least 20 mmHg or diastolic blood pressure of at least 10 mmHg within 3 min of standing.
- Hypo- or hyperthyroidism (TSH <0.5 or > 5.0 mU/L), abnormal liver function (AST or ALT more than 2 times the upper limit of normal, ULN), and alteration of kidney function.
- The values of complete blood tests in out of range and abnormal values that are clinically significant as per clinical judgment.
- The recent use of psychotropic drugs (e.g., anxiolytics, hypnotics, benzodiazepines, and antidepressants) in which the dosage was not stable for 28 days before screening.
- Severe disease (requiring systemic treatment and/or hospitalization) in the last 4 weeks.
- Any other clinically significant medical condition, psychiatric condition, drug or alcohol abuse, laboratory evaluation, or abnormality that, in the opinion of the investigators, would interfere with the subject’s ability to participate in the study.
- Beck Depression Inventory-II (BDI) score > 28, indicating a severe depression that precludes the ability to exercise.
- (Only for women) State of pregnancy.
- Other disorders, injuries, diseases, or conditions that may interfere with the ability to perform exercises (e.g., history of stroke, breathing problems, traumatic brain injury, orthopedic injury, or neuromuscular disease).

Pharmacological treatments will be kept stable throughout the length of the study for all subjects involved. Participants requiring any change in PD medications will be early discontinued by the study and evaluated for their final assessments.

### 2.3 Cohort study

The primary endpoint of the METEX-PD study is the change in BDNF in PD patients performing a standardized volume of two different exercise workloads. Walsh et al. (47) highlighted the need to interpret BDNF levels considering a measurement error (up to 20%), sample age range, and characteristics. In healthy adults, basal serum BDNF is expected to range between 20 and 30 ng/mL, with an age-associated decline of at least 0.5–5% per year. Therefore, the two groups will be matched for age, sex, duration, and stage of disease (according to H&Y score) to be recruited separately and ensure comparable levels at baseline.

### 2.4 Study procedures

Following the routine clinical practice, the medical history and clinical status of PD patients admitted to intensive and non-intensive rehabilitation settings will be recorded. Patients will undergo a cardiological examination to estimate aerobic fitness, measured as maximal oxygen uptake (VO_2max_), and neuropsychological assessments, including the administration of MoCA, MMSE, and BDI-II scales.

Before starting with exercise training, all routine laboratory analyses, clinical-functional evaluations, functional-motor assessments—including movement analysis with wearable inertial sensor for kinematic parameters (BTS G-WALK system, BTS Bioengineering S.p.A, Italy)—and neuroimaging analysis (EEG and fMRI) will be performed (Table 1, T0). Biological specimens (blood, urine, and fecal samples) will be collected and appropriately stored for biobanking and research activities.

**Table 1:** Project timeline.

Activity		Screening	T0	T1	FU-1	FU-2
Study presentation		X				
Informed consent		X				
Eligibility criteria		X				
Demographic, anthropometric, and vital signs		X				
Medical history		X				
Physical/neurological examination		X				
Clinical-functional evaluations	Montreal Cognitive Assessment (MoCA)	X	X	X	X	X
	Mini-Mental State Examination (MMSE)	X	X	X	X	X
	Beck Depression Inventory II (BDI-II)	X		X	X	X
	Wearing OFF Questionnaire-19 (WOF-19)		X	X	X	X
	Non-Motor Symptoms Scale (NMSS)		X	X	X	X
	Frontal Assessment Battery (FAB)		X	X	X	X
	O’clock Drawing Test		X	X	X	X
	Parkinson’s Disease Questionnaire-39 (PDQ-39)		X	X	X	X
Functional-motor assessments	Hoehn and Yahr (H&Y)	X	X	X	X	X
	MDS—Unified Parkinson’s Disease Rating Scale (MDS-UPDRS)		X	X	X	X
	Gait Analysis		X	X	X	X
	Time Up and Go (TUG)		X	X	X	X
	6-min Walk Test (6-MWT)		X	X	X	X
	Berg Balance Scale (BBS)		X	X	X	X
Blood samples	Plasma EDTA		X	X	X	X
	Serum		X	X	X	X
	Fecal sample		X	X		
Functional neuroimaging	EEG		X	X		X
	fMRI		X	X		X

Schedule of activities and list of assessments across the study timepoints. T0: baseline; T1: end of a 4-week exercise training program; FU-1: follow-up assessments at week 8; FU-2: follow-up assessment at week 12; EDTA: 2,2′,2″,2″′-(ethane-1,2-diyldinitrilo)tetraacetic acid; EEG: electroencephalogram; fMRI: functional magnetic resonance imaging.

A recent systematic review and network meta-analysis by Zhou et al. (48) coded the intensity and period of aerobic and resistance exercise applied in clinical trials involving people with PD, following the recommendations of ACSM (3). Based on these considerations, the exercise parameters will be set for intensive and non-intensive rehabilitation settings.

#### 2.4.1 Standardization of exercise volume: the metabolic cost of exercise

The study will compare the exercise volume of two different rehabilitation settings, measured as METs-minutes/week. MET intensity will be set according to the recently updated 2024 Adult Compendium of Physical Activities (44). The use of MET as an intensity index enables us to compare different types of exercises in terms of energy expenditure: for example, walking on a treadmill at 4.0–4.4 mph (6.4 to 7.1 km/h) with 0% grade consumes 5.8 METs and stationary cycling at 70–80 watts (44). In this regard, patients enrolling in this clinical trial could be trained alternatively with the treadmill or stationary bike. However, treadmill use leads to a more normalized gait pattern compared with cycle training, improving clinically relevant gait parameters, such as gait speed and stride length (49, 50) and will be adopted in the METEX-PD trial.

In detail, PD patients of the non-intensive rehabilitation group will perform a 45-min daily session of low-intensity aerobic exercise of 2–3 METs [37–45% VO_2max_; 57–63% HR_max_ according to Zhou et al. (48)] twice a week, for 4 weeks. Therefore, the volume of exercise for a participant of the non-intensive group—with a standard weight of 70 kg—who will exercise on the treadmill walking at ~2 km/h at 0% grade for 45 min, twice a week, will be calculated as follows:


(2METs∗45min∗2sessions)=180METs−min/week

Similarly, PD patients of the intensive rehabilitation group will exercise for 45 min daily at high-intensity aerobic training of 6–8.8 METs [46–91% VO_2max_; 76–95% HR_max_ according to Zhou et al. (48)], 5 days per week, for 4 weeks. For the intensive group, the exercise workload will be at least as follows:


(6METs∗45min∗5sessions)=1350METs−min/week

**Figure 3:**
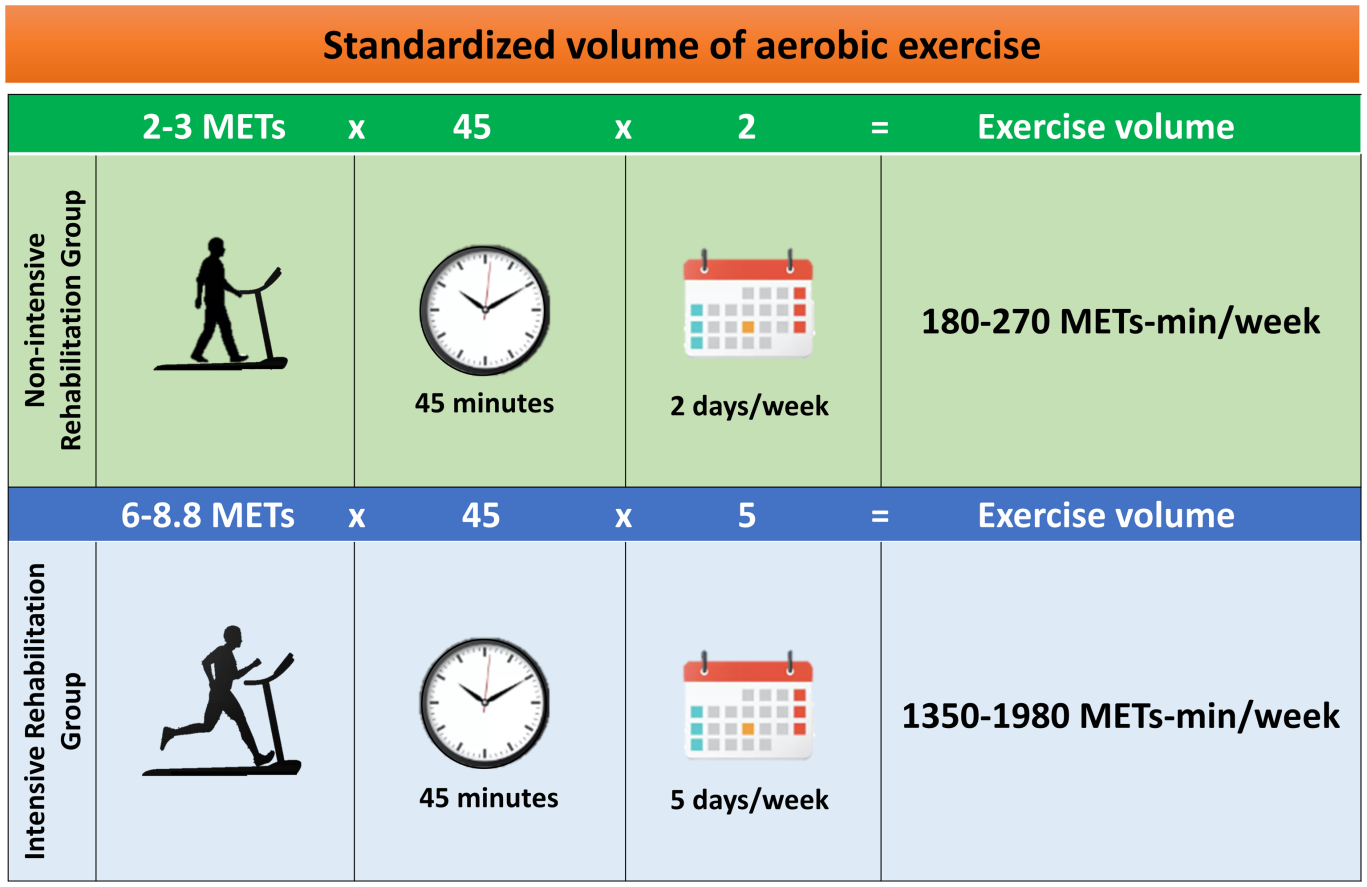
Standardization of exercise volume in terms of metabolic equivalent of tasks (METs)-min/week (3). Each group will exercise with a defined frequency, intensity, and duration.

Over the first week, the exercise professionals will set an incremental exercise to reach the predetermined intensity. Each session of aerobic exercise in both groups will include an additional 10-min warm-up and 5-min cool-down. Furthermore, the assessment of lactate concentration as an internal load marker of exercise intensity will ensure to study in aerobic conditions under the lactate threshold (<4 mM lactate) throughout the training period (Figure 3).

#### 2.4.2 Biospecimen sampling for biomolecular analysis

Following the best clinical practices, blood samples will be collected at baseline and at the end (4 weeks), 1 h after the first and last sessions of the exercise program for routine hematological and chemical analysis. In detail, the procedures require the collection of 12 mL of blood in ethylenediaminetetraacetic acid (EDTA) tubes for plasma, another 10 mL of serum in serum collection tubes, a urinary sample for urinalysis, and fecal samples for additional analysis. All samples will be marked with a unique participant code and processed to be aliquoted and stored at −20°C or − 80°C for future analysis.

The collection of biological samples will be performed during 8-week (FU-1) and 12-week (FU-2) outpatient visits, which patients regularly undergo to ensure proper continuity of care.

### 2.5 Primary outcomes

The primary outcome of the METEX-PD study is the change in BDNF assessed in peripheral blood samples (ng/ml) in PD patients performing two different standardized volumes (METs-min/week) of physical exercise in routine clinical practice.

### 2.6 Secondary outcomes

The secondary outcomes of the METEX-PD study are changes in biochemical parameters, functional-motor assessments, clinical-functional evaluations, and brain imaging.

- *Biochemical analysis will be assessed as follows:*

i Change in IGF-1 assessed by peripheral blood samples (μg/L);
ii Change in FNDC5/irisin by peripheral blood samples (ng/ml);
iii Change in high sensitivity C-reactive protein (CRP) assessed by peripheral blood samples (mg/L);
iv Change in platelet distribution width (PDW) and number of platelets assessed by peripheral blood samples;
v Change in blood lactate levels (mM) assessed using finger-stick capillary blood samples;
vi Change in gut microbial diversity (species diversity %) assessed by next-generation sequencing (NGS) of the V3–V4 region of the 16S rDNA gene.

- *Functional-motor assessments will be evaluated as follows:*

vii Change in Movement Disorder Society—Unified Parkinson’s Disease Rating Scale (MDS-UPDRS) part II (motor symptoms of daily living), part III (motor examination), and part IV (motor complications), a commonly used tool to measure the disease progression (51);
viii Change in clinical evaluation of walking speed and gait-related spatiotemporal parameters by using a wearable inertial sensor device (G-sensor, BTS Bioengineering, Milan) (52):

- Stride length [m], the distance between two consecutive hell strikes of the same foot;
- Stride length/height [%], the stride length normalized by subject height;
- Cadence [steps/min], the number of steps in a minute;
- Propulsion [m/ss], the anterior–posterior acceleration peak during the lower limb swing phase;

ix Change in execution timing of Time Up and Go, a reliable and valid test for assessing mobility, balance, walking ability, and fall risk (53);
x Change in functional capacity evaluated by a 6-min Walking Test (6MWT), a standardized method to assess the maximal patient’s capacity to walk as far as possible (measured in meters) (54);
xi Change in Berg Balance Scale (BBS), which is a widely used clinical test to assess static and dynamic balance abilities (55).

- *Clinical-functional evaluations will be assessed as follows:*

xii Change in cognitive function through the administration of MMSE, MoCA, Frontal Assessment Battery (FAB), and clock-drawing tests, which are widely used to assess multiple cognitive domains (56–58);
xiii Change in major depression symptoms will be evaluated using the BDI-II scale, a 21-question multiple-choice self-report inventory psychometric test, for measuring the severity of depression (59).
xiv Change in non-motor symptoms will be assessed, in addition to the MDS-UPDRS part I scale related to non-motor symptoms of daily living, by using the Non-Motor Symptoms Scale (NMSS) in PD (60);
xv Variation in wearing OFF episodes will be assessed by Wearing OFF Questionnaire-19 (WOQ-19) administration (61);
xvi Change in quality of life will be measured using the PDQ-39 questionnaire, which assesses how often people living with PD experience difficulties across eight dimensions of daily living (62).

- *Brain remodeling will be evaluated through functional neuroimaging:*

xvii Changes in the cortical activity measured with resting-state electroencephalography (rsEEG) (63);
xviii Changes in brain connectivity through functional magnetic resonance imaging (fMRI) exploring variation in the hippocampus, basal ganglia, cerebellum, and areas of sensory-motor integrations involved in the movement.

### 2.7 Participant timeline

The participant timeline is presented in Table 1.

### 2.8 Data management

Long-term data sharing and retention is envisaged to store and make publicly available data beyond the project lifetime. All procedures provided by this study will be performed following all the authorizations required by local law (D.Lgs. 196/2003). Each subject will be identified by a unique code throughout the study.

### 2.9 Statistical analysis

All outcome measures will be tested to assess whether they follow a normal distribution. The graphical evaluation method with normal probability plot and the Shapiro–Wilk and Kolmogorov–Smirnov tests will be used to choose the most appropriate statistical test (parametric or non-parametric tests). Descriptive statistics of all variables will be evaluated. The central tendency will be calculated as mean ± standard deviation (SD), or median with percentile ranks, depending on the nature of the variables. If data will be normally distributed, the difference between pre- and post-exercise training programs within the groups (Group 1: non-intensive rehabilitation and Group 2: intensive rehabilitation) will be evaluated using Student’s *t*-test for paired data. Differences between groups will be evaluated using the Student’s *t*-test for unpaired data. If data do not meet the normal distribution, Wilcoxon and Mann–Whitney non-parametric tests will be used.

Repeated-measures analysis of variance (ANOVA) will be used to compare the changes or delta changes (pre−/post-exercise training program/FU-1/FU-2) over time (2 groups x 4 times mixed model ANOVA, within factor). In case of significant time, group, or interaction effects, *post-hoc* pairwise comparisons will be conducted using Tukey’s HSD correction for multiple pairwise comparisons. Otherwise, the Kruskal–Wallis non-parametric test will be used when variables do not meet normal distribution. The level of significance is set at a *p*-value of <0.05.

### 2.10 Ethics approval and consent to participate

The METEX-PD study has been approved by the local ethics committee (no Protocol 0107243/2023) and will be conducted in accordance with the Declaration of Helsinki. Participants will be included in the study after signing informed consent.

The trial has been registered on ClinicalTrials.gov with the identification number: NCT06339398.

## 3 Discussion

The current estimates show a PD pandemic trend driven primarily by aging, projecting the number of patients to be over 12 million by 2040 (64). Despite the intense research efforts in pharmacological, rehabilitative, and surgical fields, PD remains a chronic neurodegenerative disease, lacking a disease-modifying therapy. Systematic review and meta-analysis supported the efficacy of physical activity and exercise, reflecting a growing interest in the promotion of rehabilitation programs as a disease-modifying treatment for PD (18, 19).

The Physical Activity Guidelines Advisory Committee affirmed that *“the health benefits of physical activity depend mainly on total weekly energy expenditure due to physical activity”* and concluded that a dose–response relationship exists between physical activity and health benefits. Therefore, the recommendation is to achieve ≥500 METs-min/week of physical activity for health benefits and additional benefits when achieving ≥1,000 METs-min/week (65).

Although the 2018 US updated guidelines (66) and WHO Guidelines on Physical Activity and Sedentary Behavior (67) recommend at least 150–300 min/week of moderate aerobic exercise (3 to 5.9 MET) or 75–150 min/week of vigorous (≥ 6 MET) aerobic exercise for older adults (> 65+ years), the majority of healthy older adults do not achieve these weekly minimal activity levels (68). Following a diagnosis of PD, activity levels drop under the recommended volume of physical activity and are significantly lower than those of healthy peers (69, 70).

The dose–response relationship, proposed by the 2008 Physical Activity Guidelines Advisory Committee, reflects the rationale of the METEX-PD trial. In other terms, the standardization of weekly energy expenditure due to exercise is the prerequisite to prescribing exercise as medicine with a patient-specific “dose.”

Similar to the pharmacological pipeline, once exercise safety is established (5), the efficacy of different dosages of exercise volume needs to be further elucidated, investigating the molecular determinants driving the known physical activity benefits on motor function and quality of life in people with PD (1).

Starting from the biochemistry definition of energy expenditure, which requires oxygen consumption to generate ATP during the workout—and therefore setting aerobic fitness and monitoring the workload through lactate measuring—this observational study will enable us to compare the disease-modifying effects of two rehabilitation settings in routine clinical practice. The comparison of two different “dosages” of exercise will provide the preliminary evidence to clarify the dose–response relationship between different volumes of exercise, and therefore different amounts of energy expenditure, and the induction of disease-modifying mechanisms.

The METEX-PD study will focus on the identification and change of peripheral biomarkers, leading to the preliminary evidence of the molecular basis underlying the disease modification process through functional-motor assessments, clinical-functional evaluations, and functional neuroimaging.

In this regard, the METEX-PD study will especially focus on the contribution of BDNF as a driver of neuroplasticity in PD patients performing physical exercise, considering both central and peripheral sources of this neurotrophic factor. Indeed, although the brain is the primary source of BDNF during exercise, the contribution of peripheral sources deserves to be elucidated, considering primarily the role of skeletal muscle, platelets, and PBMCs.

Emerging evidence from animal studies revealed that skeletal muscle contraction can increase concentrations of BDNF levels in the central nervous system, through direct and indirect mechanisms. The release of myokines, such as irisin, induces BDNF through a peroxisome proliferator-activated receptor-gamma coactivator (PGC)-1alpha/fibronectin type III domain-containing protein 5 (PGC-1α/FNDC5) pathway in the hippocampus of mice performing endurance exercise (38); energy consumption to sustain muscle contraction induces an increase in circulating blood lactate, which induces the *Bdnf* gene expression and tropomyosin receptor kinase-B (TrkB) signaling in the hippocampus via nicotinamide adenine dinucleotide (NAD)-dependent deacetylase sirtuin-1 (SIRT1). In turn, SIRT1 increases the levels of the PGC-1α/FNDC5 pathway and thus the *bdnf* expression (39).

Instead, the peripheral increase could be the result of BDNF release from platelets, as these cells can store the majority of pro-BDNF, which is supposed to be produced in megakaryocytes (40–42), and secreted in its mature form in a dose-dependent manner after shear stress, such as being induced by exercise (71). In addition to platelets, the involvement of PBMCs cannot be excluded as revealed by an increase in the *bdnf* expression during exercise in a dose-dependent response (72). Finally, it is worth noting the contribution of vascular endothelial cells to the production, storage, and release of BDNF centrally and peripherally, in response to endothelial nitric oxide synthase (eNOS) activity (43).

## 4 Limitations

There are some limitations to this study. Exercise-induced energy expenditure (ExEE) represents only ~15–30% of total energy expenditure (TEE), which includes also the energy costs to sustain the basal metabolic rate (BMR, 60–80% of TEE) and the energy required to absorb and process food for storage (thermic effect of food, TEF, ~10% of TEE) (73, 74). As a pilot study, METEX-PD will focus solely on the costs of energy expenditure due to exercise, although PD patients generally follow a regular diet regimen that does not interfere with their pharmacological therapies.

Moreover, this is a monocentric clinical trial, in which, findings will have to be further validated. However, due to a paucity of results on the dose–response effects (which volume of exercise is sufficient to be effective in inducing a change in NFs?) and in the absence of clear information on the most representative peripheral biomarkers (BDNF? IGF-1? irisin? Change in PDW?) of exercise dose efficacy, the conduction of a monocentric pilot study is strictly necessary to evaluate the feasibility of a multicenter clinical trial.

Therefore, the preliminary results of the pilot METEX-PD clinical trial will represent the prerequisite for the development of a rigorous multicenter randomized controlled trial with an appropriate sample size to investigate the molecular mechanisms of selected NFs, such as BDNF, IGF-1, or irisin, driving the disease-modifying effects in people with PD performing a defined dosage of structured exercise.

Finally, this trial is a multimodal clinical, biological, and functional neuroimaging study with an evident translational value. The results of this study can improve the clinical management of PD patients by addressing the most effective aerobic exercise in different rehabilitation settings. The effects of standardized training programs will be demonstrated not only by clinical evaluation but also by changes in biological markers and neuroimaging. The study will provide an innovation in highlighting markers that can accurately determine the effectiveness of rehabilitation treatments.

## Author contributions

RR: Writing – review & editing, Writing – original draft, Methodology, Investigation, Formal analysis, Data curation. EP: Writing – review & editing, Methodology. GA: Writing – review & editing, Methodology. MGu: Writing – review & editing, Methodology. SD-Z: Writing – review & editing, Methodology. MGo: Writing – review & editing. VS: Writing – review & editing. FS: Writing – review & editing, Writing – original draft, Supervision, Project administration, Conceptualization. MP: Writing – review & editing, Writing – original draft, Supervision, Project administration, Conceptualization.

## References

[1] Ernst M Folkerts AK Gollan R Lieker E Caro-Valenzuela J Adams A . Physical exercise for people with Parkinson's disease: a systematic review and network meta-analysis. Cochrane Database Syst Rev. (2024) 4:CD013856. doi: 10.1002/14651858.CD013856.pub3, PMID: 38588457 PMC11001292

[2] Alberts JL Rosenfeldt AB. The universal prescription for Parkinson's disease: exercise. J Parkinsons Dis. (2020) 10:S21–7. doi: 10.3233/JPD-202100, PMID: 32925109 PMC7592674

[3] Garber CE Blissmer B Deschenes MR Franklin BA Lamonte MJ Lee IM . American College of Sports Medicine position stand. Quantity and quality of exercise for developing and maintaining cardiorespiratory, musculoskeletal, and neuromotor fitness in apparently healthy adults: guidance for prescribing exercise. Med Sci Sports Exerc. (2011) 43:1334–59. doi: 10.1249/MSS.0b013e318213fefb, PMID: 21694556

[4] Caspersen CJ Powell KE Christenson GM. Physical activity, exercise, and physical fitness: definitions and distinctions for health-related research. Public Health Rep. (1985) 100:126–31. PMID: 3920711 PMC1424733

[5] Schenkman M Moore CG Kohrt WM Hall DA Delitto A Comella CL . Effect of high-intensity treadmill exercise on motor symptoms in patients with De novo Parkinson disease: a phase 2 randomized clinical trial. JAMA Neurol. (2018) 75:219–26. doi: 10.1001/jamaneurol.2017.3517, PMID: 29228079 PMC5838616

[6] Patterson CG Joslin E Gil AB Spigle W Nemet T Chahine L . Study in Parkinson's disease of exercise phase 3 (SPARX3): study protocol for a randomized controlled trial. Trials. (2022) 23:855. doi: 10.1186/s13063-022-06703-0, PMID: 36203214 PMC9535216

[7] Tillerson JL Caudle WM Reverón ME Miller GW. Exercise induces behavioral recovery and attenuates neurochemical deficits in rodent models of Parkinson's disease. Neuroscience. (2003) 119:899–911. doi: 10.1016/S0306-4522(03)00096-4, PMID: 12809709

[8] Petzinger GM Fisher B Hogg E Abernathy A Arevalo P Nixon K . Behavioral motor recovery in the 1-methyl-4-phenyl-1,2,3,6-tetrahydropyridine-lesioned squirrel monkey (*Saimiri sciureus*): changes in striatal dopamine and expression of tyrosine hydroxylase and dopamine transporter proteins. J Neurosci Res. (2006) 83:332–47. doi: 10.1002/jnr.20730, PMID: 16385585

[9] Petzinger GM Walsh JP Akopian G Hogg E Abernathy A Arevalo P . Effects of treadmill exercise on dopaminergic transmission in the 1-methyl-4-phenyl-1,2,3,6-tetrahydropyridine-lesioned mouse model of basal ganglia injury. J Neurosci. (2007) 27:5291–300. doi: 10.1523/JNEUROSCI.1069-07.2007, PMID: 17507552 PMC6672356

[10] Zigmond MJ Cameron JL Leak RK Mirnics K Russell VA Smeyne RJ . Triggering endogenous neuroprotective processes through exercise in models of dopamine deficiency. Parkinsonism Relat Disord. (2009) 15:S42–5. doi: 10.1016/S1353-8020(09)70778-3, PMID: 20083005

[11] Petzinger GM Fisher BE Van Leeuwen JE Vukovic M Akopian G Meshul CK . Enhancing neuroplasticity in the basal ganglia: the role of exercise in Parkinson's disease. Mov Disord. (2010) 25 Suppl 1:S141–5. doi: 10.1002/mds.22782 20187247 PMC4111643

[12] Vučković MG Li Q Fisher B Nacca A Leahy RM Walsh JP . Exercise elevates dopamine D2 receptor in a mouse model of Parkinson's disease: in vivo imaging with [^18^F]fallypride. Mov Disord. (2010) 25:2777–84. doi: 10.1002/mds.23407, PMID: 20960487 PMC3273304

[13] Zhang X Xu S Hu Y Liu Q Liu C Chai H . Irisin exhibits neuroprotection by preventing mitochondrial damage in Parkinson's disease. NPJ Parkinsons Dis. (2023) 9:13. doi: 10.1038/s41531-023-00453-9, PMID: 36720890 PMC9889817

[14] Tajiri N Yasuhara T Shingo T Kondo A Yuan W Kadota T . Exercise exerts neuroprotective effects on Parkinson's disease model of rats. Brain Res. (2010) 1310:200–7. doi: 10.1016/j.brainres.2009.10.075, PMID: 19900418

[15] Marino G Campanelli F Natale G De Carluccio M Servillo F Ferrari E . Intensive exercise ameliorates motor and cognitive symptoms in experimental Parkinson's disease restoring striatal synaptic plasticity. Sci Adv. (2023) 9:eadh1403. doi: 10.1126/sciadv.adh1403, PMID: 37450585 PMC10348672

[16] Fisher BE Li Q Nacca A Salem GJ Song J Yip J . Treadmill exercise elevates striatal dopamine D2 receptor binding potential in patients with early Parkinson's disease. Neuroreport. (2013) 24:509–14. doi: 10.1097/WNR.0b013e328361dc13, PMID: 23636255

[17] Johansson ME Cameron IGM Van der Kolk NM de Vries NM Klimars E Toni I . Aerobic exercise alters brain function and structure in Parkinson's disease: a randomized controlled trial. Ann Neurol. (2022) 91:203–16. doi: 10.1002/ana.26291, PMID: 34951063 PMC9306840

[18] Kaagman DGM van Wegen EEH Cignetti N Rothermel E Vanbellingen T Hirsch MA. Effects and mechanisms of exercise on brain-derived neurotrophic Factor (BDNF) levels and clinical outcomes in people with Parkinson's disease: a systematic review and Meta-analysis. Brain Sci. (2024) 14:194. doi: 10.3390/brainsci14030194, PMID: 38539583 PMC10968162

[19] Rotondo R Proietti S Perluigi M Padua E Stocchi F Fini M . Physical activity and neurotrophic factors as potential drivers of neuroplasticity in Parkinson's disease: a systematic review and meta-analysis. Ageing Res Rev. (2023) 92:102089. doi: 10.1016/j.arr.2023.102089, PMID: 37844764

[20] Gerecke KM Jiao Y Pani A Pagala V Smeyne RJ. Exercise protects against MPTP-induced neurotoxicity in mice. Brain Res. (2010) 1341:72–83. doi: 10.1016/j.brainres.2010.01.053, PMID: 20116369 PMC2884060

[21] Real CC Doorduin J Kopschina Feltes P Vállez García D de Paula FD Britto LR . Evaluation of exercise-induced modulation of glial activation and dopaminergic damage in a rat model of Parkinson's disease using. J Cereb Blood Flow Metab. (2019) 39:989–1004. doi: 10.1177/0271678X17750351, PMID: 29271291 PMC6545619

[22] Binda KH Lillethorup TP Real CC Bærentzen SL Nielsen MN Orlowski D . Exercise protects synaptic density in a rat model of Parkinson's disease. Exp Neurol. (2021) 342:113741. doi: 10.1016/j.expneurol.2021.113741, PMID: 33965411

[23] Ferreira AFF Binda KH Real CC. The effects of treadmill exercise in animal models of Parkinson's disease: a systematic review. Neurosci Biobehav Rev. (2021) 131:1056–75. doi: 10.1016/j.neubiorev.2021.10.019, PMID: 34688727

[24] de Laat B Hoye J Stanley G Hespeler M Ligi J Mohan V . Intense exercise increases dopamine transporter and neuromelanin concentrations in the substantia nigra in Parkinson's disease. NPJ Parkinsons Dis. (2024) 10:34. doi: 10.1038/s41531-024-00641-1 38336768 PMC10858031

[25] Liu PZ Nusslock R. Exercise-mediated neurogenesis in the Hippocampus via BDNF. Front Neurosci. (2018) 12:52. doi: 10.3389/fnins.2018.00052, PMID: 29467613 PMC5808288

[26] Vaynman S Ying Z Gomez-Pinilla F. Hippocampal BDNF mediates the efficacy of exercise on synaptic plasticity and cognition. Eur J Neurosci. (2004) 20:2580–90. doi: 10.1111/j.1460-9568.2004.03720.x, PMID: 15548201

[27] Vaynman SS Ying Z Yin D Gomez-Pinilla F. Exercise differentially regulates synaptic proteins associated to the function of BDNF. Brain Res. (2006) 1070:124–30. doi: 10.1016/j.brainres.2005.11.062, PMID: 16413508

[28] Cotman CW Berchtold NC Christie LA. Exercise builds brain health: key roles of growth factor cascades and inflammation. Trends Neurosci. (2007) 30:464–72. doi: 10.1016/j.tins.2007.06.011, PMID: 17765329

[29] Frazzitta G Maestri R Ghilardi MF Riboldazzi G Perini M Bertotti G . Intensive rehabilitation increases BDNF serum levels in parkinsonian patients: a randomized study. Neurorehabil Neural Repair. (2014) 28:163–8. doi: 10.1177/1545968313508474, PMID: 24213955

[30] Szymura J Kubica J Wiecek M Pera J. The Immunomodulary effects of systematic exercise in older adults and people with Parkinson's disease. J Clin Med. (2020) 9:184. doi: 10.3390/jcm9010184, PMID: 31936624 PMC7019419

[31] O'Callaghan A Harvey M Houghton D Gray WK Weston KL Oates LL . Comparing the influence of exercise intensity on brain-derived neurotrophic factor serum levels in people with Parkinson's disease: a pilot study. Aging Clin Exp Res. (2020) 32:1731–8. doi: 10.1007/s40520-019-01353-w, PMID: 31606860

[32] Freidle M Johansson H Ekman U Lebedev AV Schalling E Thompson WH . Behavioural and neuroplastic effects of a double-blind randomised controlled balance exercise trial in people with Parkinson's disease. NPJ Parkinsons Dis. (2022) 8:12. doi: 10.1038/s41531-021-00269-5, PMID: 35064138 PMC8782921

[33] Li JA Loevaas MB Guan C Goh L Allen NE Mak MKY . Does exercise attenuate disease progression in people with Parkinson's disease? A systematic review with Meta-analyses. Neurorehabil Neural Repair. (2023) 37:328–52. doi: 10.1177/15459683231172752, PMID: 37166181 PMC10272626

[34] Sacheli MA Neva JL Lakhani B Murray DK Vafai N Shahinfard E . Exercise increases caudate dopamine release and ventral striatal activation in Parkinson's disease. Mov Disord. (2019) 34:1891–900. doi: 10.1002/mds.27865, PMID: 31584222

[35] Albrecht F Pereira JB Mijalkov M Freidle M Johansson H Ekman U . Effects of a highly challenging balance training program on motor function and brain structure in Parkinson's disease. J Parkinsons Dis. (2021) 11:2057–71. doi: 10.3233/JPD-212801, PMID: 34511513 PMC8673526

[36] King LA Mancini M Smulders K Harker G Lapidus JA Ramsey K . Cognitively challenging agility boot camp program for freezing of gait in Parkinson disease. Neurorehabil Neural Repair. (2020) 34:417–27. doi: 10.1177/1545968320909331, PMID: 32249668 PMC7217755

[37] Contrepois K Wu S Moneghetti KJ Hornburg D Ahadi S Tsai MS . Molecular choreography of acute exercise. Cell. (2020) 181:1112–30.e16. doi: 10.1016/j.cell.2020.04.043 32470399 PMC7299174

[38] Wrann CD White JP Salogiannnis J Laznik-Bogoslavski D Wu J Ma D . Exercise induces hippocampal BDNF through a PGC-1α/FNDC5 pathway. Cell Metab. (2013) 18:649–59. doi: 10.1016/j.cmet.2013.09.008, PMID: 24120943 PMC3980968

[39] El Hayek L Khalifeh M Zibara V Abi Assaad R Emmanuel N Karnib N . Lactate mediates the effects of exercise on learning and memory through SIRT1-dependent activation of hippocampal brain-derived neurotrophic Factor (BDNF). J Neurosci. (2019) 39:2369–82. doi: 10.1523/JNEUROSCI.1661-18.2019, PMID: 30692222 PMC6435829

[40] Yamamoto H Gurney ME. Human platelets contain brain-derived neurotrophic factor. J Neurosci. (1990) 10:3469–78. doi: 10.1523/JNEUROSCI.10-11-03469.1990, PMID: 2230938 PMC6570101

[41] Chacón-Fernández P Säuberli K Colzani M Moreau T Ghevaert C Barde YA. Brain-derived neurotrophic Factor in megakaryocytes. J Biol Chem. (2016) 291:9872–81. doi: 10.1074/jbc.M116.720029, PMID: 27006395 PMC4858990

[42] Radka SF Holst PA Fritsche M Altar CA. Presence of brain-derived neurotrophic factor in brain and human and rat but not mouse serum detected by a sensitive and specific immunoassay. Brain Res. (1996) 709:122–30. doi: 10.1016/0006-8993(95)01321-0 8869564

[43] Monnier A Prigent-Tessier A Quirié A Bertrand N Savary S Gondcaille C . Brain-derived neurotrophic factor of the cerebral microvasculature: a forgotten and nitric oxide-dependent contributor of brain-derived neurotrophic factor in the brain. Acta Physiol (Oxf). (2017) 219:790–802. doi: 10.1111/apha.12743 27364224

[44] Herrmann SD Willis EA Ainsworth BE Barreira TV Hastert M Kracht CL . 2024 adult compendium of physical activities: a third update of the energy costs of human activities. J Sport Health Sci. (2024) 13:6–12. doi: 10.1016/j.jshs.2023.10.010, PMID: 38242596 PMC10818145

[45] Gibb WR Lees AJ. The relevance of the Lewy body to the pathogenesis of idiopathic Parkinson's disease. J Neurol Neurosurg Psychiatry. (1988) 51:745–52. doi: 10.1136/jnnp.51.6.745, PMID: 2841426 PMC1033142

[46] Hoehn MM Yahr MD. Parkinsonism: onset, progression and mortality. Neurology. (1967) 17:427–42. doi: 10.1212/WNL.17.5.427 6067254

[47] Walsh EI Smith L Northey J Rattray B Cherbuin N. Towards an understanding of the physical activity-BDNF-cognition triumvirate: a review of associations and dosage. Ageing Res Rev. (2020) 60:101044. doi: 10.1016/j.arr.2020.101044 32171785

[48] Zhou X Zhao P Guo X Wang J Wang R. Effectiveness of aerobic and resistance training on the motor symptoms in Parkinson's disease: systematic review and network meta-analysis. Front Aging Neurosci. (2022) 14:935176. doi: 10.3389/fnagi.2022.935176, PMID: 35978948 PMC9376630

[49] Mehrholz J Kugler J Storch A Pohl M Elsner B Hirsch K. Treadmill training for patients with Parkinsons disease. Cochrane Database Syst Rev. (2015) 8:CD007830. doi: 10.1002/14651858.CD007830.pub3 26297797

[50] Fernández-Del-Olmo M. Treadmill vs cycling in Parkinson's disease rehabilitation: commentary on intensive cycle ergometer training improves gait speed and endurance in patients with Parkinson's disease: a comparison with treadmill training by Arcolin et al., 2016. Restor Neurol Neurosci. (2016) 34:691–2. doi: 10.3233/RNN-160648, PMID: 27472846

[51] Goetz CG Fahn S Martinez-Martin P Poewe W Sampaio C Stebbins GT . Movement Disorder Society-sponsored revision of the unified Parkinson's disease rating scale (MDS-UPDRS): process, format, and clinimetric testing plan. Mov Disord. (2007) 22:41–7. doi: 10.1002/mds.21198 17115387

[52] Zago M Sforza C Pacifici I Cimolin V Camerota F Celletti C . Gait evaluation using inertial measurement units in subjects with Parkinson's disease. J Electromyogr Kinesiol. (2018) 42:44–8. doi: 10.1016/j.jelekin.2018.06.009 29940494

[53] Kleiner AFR Pacifici I Vagnini A Camerota F Celletti C Stocchi F . Timed up and go evaluation with wearable devices: validation in Parkinson's disease. J Bodyw Mov Ther. (2018) 22:390–5. doi: 10.1016/j.jbmt.2017.07.006, PMID: 29861240

[54] Üğüt BO Kalkan AC Kahraman T Dönmez Çolakoğlu B Çakmur R Genç A. Determinants of 6-minute walk test in people with Parkinson's disease. Ir J Med Sci. (2023) 192:359–67. doi: 10.1007/s11845-022-02954-7 35199303

[55] Qutubuddin AA Pegg PO Cifu DX Brown R McNamee S Carne W. Validating the berg balance scale for patients with Parkinson's disease: a key to rehabilitation evaluation. Arch Phys Med Rehabil. (2005) 86:789–92. doi: 10.1016/j.apmr.2004.11.005, PMID: 15827933

[56] Folstein MF Folstein SE McHugh PR. "Mini-mental state". A practical method for grading the cognitive state of patients for the clinician. J Psychiatr Res. (1975) 12:189–98. doi: 10.1016/0022-3956(75)90026-6 1202204

[57] Nasreddine ZS Phillips NA Bédirian V Charbonneau S Whitehead V Collin I . The Montreal cognitive assessment, MoCA: a brief screening tool for mild cognitive impairment. J Am Geriatr Soc. (2005) 53:695–9. doi: 10.1111/j.1532-5415.2005.53221.x, PMID: 15817019

[58] Bezdicek O Růžička F Fendrych Mazancova A Roth J Dušek P Mueller K . Frontal assessment battery in Parkinson's disease: validity and morphological correlates. J Int Neuropsychol Soc. (2017) 23:675–84. doi: 10.1017/S1355617717000522, PMID: 28716165

[59] Beck AT Steer RA Brown GK. BDI-II manual. San Antonio, TX: The Psychological Corporation (1996).

[60] van Wamelen DJ Martinez-Martin P Weintraub D Schrag A Antonini A Falup-Pecurariu C . The non-motor symptoms scale in Parkinson's disease: validation and use. Acta Neurol Scand. (2021) 143:3–12. doi: 10.1111/ane.13336 32813911

[61] Stocchi F Antonini A Barone P Tinazzi M Zappia M Onofrj M . Early DEtection of wEaring off in Parkinson disease: the DEEP study. Parkinsonism Relat Disord. (2014) 20:204–11. doi: 10.1016/j.parkreldis.2013.10.027, PMID: 24275586

[62] Jenkinson C Fitzpatrick R Peto V Greenhall R Hyman N. The Parkinson's disease questionnaire (PDQ-39): development and validation of a Parkinson's disease summary index score. Age Ageing. (1997) 26:353–7. doi: 10.1093/ageing/26.5.353, PMID: 9351479

[63] Babiloni C Del Percio C Lizio R Noce G Lopez S Soricelli A . Levodopa may affect cortical excitability in Parkinson's disease patients with cognitive deficits as revealed by reduced activity of cortical sources of resting state electroencephalographic rhythms. Neurobiol Aging. (2019) 73:9–20. doi: 10.1016/j.neurobiolaging.2018.08.010 30312790

[64] Dorsey ER Sherer T Okun MS Bloem BR. The emerging evidence of the Parkinson pandemic. J Parkinsons Dis. (2018) 8:S3–8. doi: 10.3233/JPD-181474, PMID: 30584159 PMC6311367

[65] Physical Activity Guidelines Advisory Committee. Physical activity guidelines advisory committee report, 2008. Washington, DC: U.S. Department of Health and Human Services (2008). 10.1111/j.1753-4887.2008.00136.x 19178654

[66] Piercy KL Troiano RP Ballard RM Carlson SA Fulton JE Galuska DA . The physical activity guidelines for Americans. JAMA. (2018) 320:2020–8. doi: 10.1001/jama.2018.14854, PMID: 30418471 PMC9582631

[67] World Health Organization, WHO guidelines on physical activity and sedentary behaviour. (2020). 33369898

[68] Hallal PC Andersen LB Bull FC Guthold R Haskell W Ekelund U . Global physical activity levels: surveillance progress, pitfalls, and prospects. Lancet. (2012) 380:247–57. doi: 10.1016/S0140-6736(12)60646-1, PMID: 22818937

[69] van Nimwegen M Speelman AD Hofman-van Rossum EJ Overeem S Deeg DJ Borm GF . Physical inactivity in Parkinson's disease. J Neurol. (2011) 258:2214–21. doi: 10.1007/s00415-011-6097-7, PMID: 21614433 PMC3225631

[70] Cavanaugh JT Ellis TD Earhart GM Ford MP Foreman KB Dibble LE. Toward understanding ambulatory activity decline in Parkinson disease. Phys Ther. (2015) 95:1142–50. doi: 10.2522/ptj.20140498, PMID: 25858971 PMC4528016

[71] Fujimura H Altar CA Chen R Nakamura T Nakahashi T Kambayashi J . Brain-derived neurotrophic factor is stored in human platelets and released by agonist stimulation. Thromb Haemost. (2002) 87:728–34. doi: 10.1055/s-0037-1613072, PMID: 12008958

[72] Brunelli A Dimauro I Sgrò P Emerenziani GP Magi F Baldari C . Acute exercise modulates BDNF and pro-BDNF protein content in immune cells. Med Sci Sports Exerc. (2012) 44:1871–80. doi: 10.1249/MSS.0b013e31825ab69b, PMID: 22543740

[73] Ravussin E Bogardus C. Relationship of genetics, age, and physical fitness to daily energy expenditure and fuel utilization. Am J Clin Nutr. (1989) 49:968–75. doi: 10.1093/ajcn/49.5.968, PMID: 2655422

[74] Westerterp KR. Physical activity and physical activity induced energy expenditure in humans: measurement, determinants, and effects. Front Physiol. (2013) 4:90. doi: 10.3389/fphys.2013.00090 23637685 PMC3636460

