# Supplementary figures and images for: Dose–response effects of physical exercise standardized volume on peripheral biomarkers, clinical response, and brain connectivity in Parkinson’s disease: a prospective, observational, cohort study
Source: Front Neurol. 2024 Jul 3;15:1412311. doi: 10.3389/fneur.2024.1412311 (PMC11251892; doi:10.3389/fneur.2024.1412311)

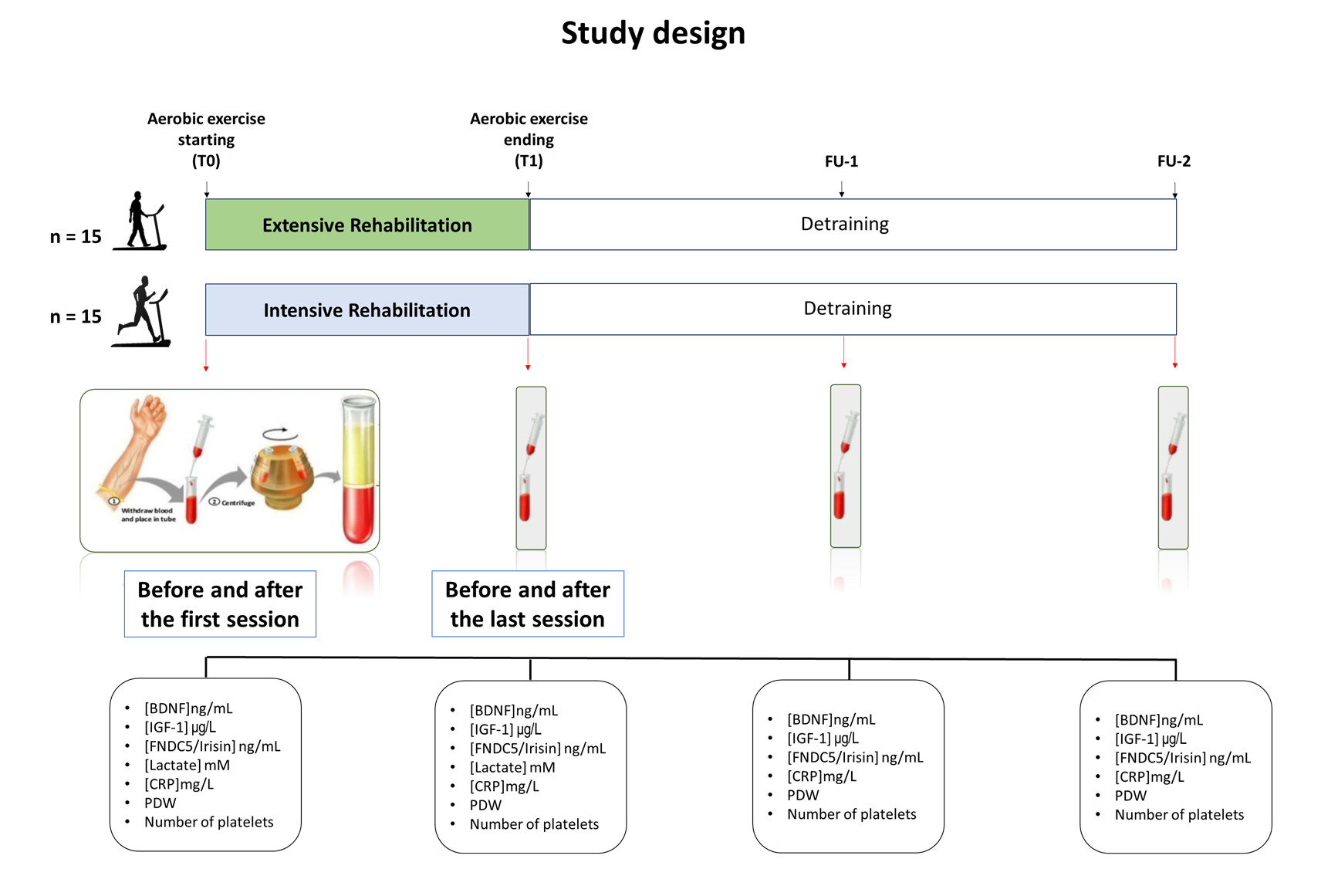

Supplement: Supplementary file 1 [file Image_1.TIF]
